# Coronary artery aneurysms occurrence risk analysis between Kawasaki disease and LRP1B gene in Taiwanese children

**DOI:** 10.7603/s40681-014-0010-5

**Published:** 2014-08-02

**Authors:** Ying-Ju Lin, Xiang Liu, Jeng-Sheng Chang, Wen- Kuei Chien, Jin-Hua Chen, Hsinyi Tsang, Chien-Hui Hung, Ting-Hsu Lin, Shao-Mei Huang, Chiu-Chu Liao, Cheng-Wen Lin, Tsung-Jung Ho, Fuu-Jen Tsai

**Affiliations:** 1 Department of Medical Research, China Medical University Hospital, Taichung, Taiwan; 2 School of Chinese Medicine, China Medical University, Taichung, Taiwan; 3 National Institute of Allergy and Infectious Diseases, National Institutes of Health, Bethesda, Maryland, USA; 4 Department of Pediatrics, China Medical University Hospital, Taichung, Taiwan; 5 Biostatistics Center, Taipei Medical University, Taichung, Taiwan; 6 Biostatistics Center, China Medical University, Taichung, Taiwan; 7 Graduate Institute of Clinical Medical Science, Chang-Gung University, Taipei, Taiwan; 8 Department of Medical Laboratory Science and Biotechnology, China Medical University, Taichung, Taiwan; 9 Division of Chinese Medicine, China Medical University Beigang Hospital, Yunlin County, Taiwan; 10 Division of Chinese Medicine, Tainan Municipal An-Nan Hospital -China Medical University, Tainan, Taiwan; 11 Department of Biotechnology, Asia University, Taichung, Taiwan

**Keywords:** KD, LRP1B, Single-nucleotide polymorphism, CAA

## Abstract

Background: Kawasaki disease (KD) is an acute and systemic vasculitis. Its complications in coronary artery aneurysms (CAA) make KD one of the leading causes of acquired cardiovascular diseases in childhood. Low density lipoprotein receptor-related protein 1B (LRP1B) is abundantly expressed in the medial layer of coronary arteries and involved in endothelium inflammations.

Purpose: We aimed to identify the role of *LRP1B* in CAA formation during KD progression.

Methods: we investigated genetic variations in LRP1B in a Taiwanese cohort of 258 KD patients (83 with CAA and 175 without CAA complications). We used univariate and multivariate regression analyses to identify the associations between *LRP1B* genetic variations and KD patients.

Results: CAA formation in KD was significantly associated with the *LRP1B* (rs6707826) genetic variant (p = 0.007). By using multivariate regression analysis, significant correlations were observed between KD with CAA complications and the presence of the TT+TG genotypes for the *LRP1B* rs6707826 single-nucleotide polymorphism (full model: odds ratio = 2.82; 95% CI = 1.33–5.78).

Conclusion: Our results suggest that genetic polymorphism of *LRP1B* gene may be used as a genetic marker for the diagnosis and prognosis of the CAA formation in KD and contribute to genetic profiling studies for personalized medicine.

## 1. Introduction

Kawasaki disease (KD) is a disease in which acute and systemic vasculitis is largely seen in infants and children under five years old [1-5]. The cause of KD is currently unknown, although there is often a pre-existing infection that may play a role in its pathogenesis. During the acute stage of KD, activation of vascular endothelial cells and increased serum levels of proinflammatory cytokines lead to inflammation and injury of blood vessels [6-8]. Coronary artery aneurysms (CAA), which is a complication during the progression of disease, makes KD one of the leading causes for acquired cardiovascular diseases in childhood.

Members of the low density lipoprotein (LDL) receptors family play a variety of roles in normal cell function and development due to their interactions with multiple ligands [9,10]. Histochemical studies have revealed that LDL receptors are markedly induced during progression of atherosclerotic lesions [11]. LDL receptors are highly expressed in smooth muscle cells (SMCs), macrophages and endothelial cells in the lesions. The low density lipoprotein receptor-related protein LRP1B is a new member of LDL receptor subgroup of this gene family. LRP1B is abundantly expressed in the medial layer of coronary arteries and to a lesser extent in thickened intimae [12]. During the acute stage of KD, activation of vascular endothelium cells with increased serum proinflammatory cytokines are involved in vessel inflammation and injury [13, 14]. Injured vascular tissues show subendothelial edema, vascular damage, gap formation, and fenestration of endothelial cells and contribute to the pathogenesis of this disorder. However, it remains unknown if LDL receptor proteins involve in the pathogenesis of KD.

We aimed to identify the role of *LRP1B* in CAA formation during KD progression by genetic association studies in a Taiwanese cohort of 258 KD patients (83 with CAA and 175 without CAA complications). The study identified *LRP1B* as a novel susceptibility locus. It is the first time to observe the association between genetic variants of LDL receptor and the CAA formation in KD and contribute to genetic profiling studies for personalized medicine [15].

## 2. Materials and methods

### 2.1. Ethical statement

This study was approved by the Human Studies Committee of China Medical University Hospital. Written informed consent was obtained from either the parents or the participants.

### 2.2. Study subjects

Unrelated individuals fulfilling the diagnostic criteria of KD (n = 258) were identified and enrolled in the study from the Department of Pediatrics at China Medical University Hospital in Taichung, Taiwan [16-20]. A total of 164 males and 94 females with an average age at diagnosis of 1.75 ± 1.61 years were included in the study. All patients were diagnosed according to KD criteria [16, 18], including fever lasting 5 days or more and at least 4 of the following symptoms: (1) changes in extremities (e.g., erythema, edema, or desquamation), (2) bilateral conjunctivitis, (3) polymorphous rash, (4) cervical lymphadenopathy, and (5) changes in lips or oral cavity (e.g., pharyngeal erythema, dry/fissured or swollen lips, strawberry tongue). All patients had regular echocardiography examinations during the acute stage, 2 months after onset, 6 months after onset, and once per year thereafter. CAA was identified when either the right coronary artery or the left coronary artery showed a dilated diameter ≥ 3 mm in children younger than 5 years of age, or ≥ 4 mm in older children [13]. Only Han Chinese individuals, who account for 98% of Taiwanese residents, were recruited. The ethnic background was assigned based on the results of the self-report questionnaires.

**Fig. 1:**
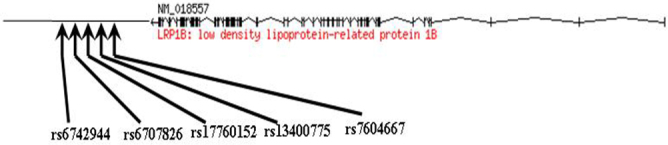
Single-nucleotide polymorphisms (SNPs) of the *LRP1B* gene used in this study. Genomic location of SNPs present on chromosome 2q22.1.

### 2.3. SNP genotyping

Five single-nucleotide polymorphisms (SNPs) from *LRP1B* were selected from the NCBI SNP database and HAPMAP website (Fig. 1 and Table 1) [21-23]. Selection criteria for including SNPs in the analysis were a minimum allele frequency of >0.05 in the Han Chinese population and Hardy–Weinberg equilibrium (HWE; *p* > 0.05). A summary of information regarding SNPs in the *LRP1B* gene (location, position, rs number, and genotype) is listed in Table 1. Briefly, genomic DNA was extracted from peripheral blood leukocytes according to standard protocols (Genomic DNA kit; Qiagen, Hilden, Germany). SNPs were genotyped using a custom-designed VeraCode GoldenGate Genotyping Assay System (Illumina) [24]; genotyping was performed as described at http://www.illumina.com/

Primers and probes were designed using Custom VeraCode GoldenGate Genotyping Assay System software. Multiplex PCRs were performed with 144-plex VeraCode SNP arrays and genotype analyses were performed using custom 96-plex SAM arrays for 96 samples. Genotype calls were automatically generated using GenCall software version 3.1.3. We assessed 8 VeraCode runs individually for intra-plate inconsistencies, such as variations in fluorescent intensities. Genotype cluster plots generated by individual VeraCode and SAM assays were visually inspected for call quality. Plots that appeared to be “unusually” clustered (i.e., those that did not match the predicted spread in terms of software-generated HWE or distance between clusters [θ]) were investigated further by selecting samples via direct Sanger sequencing for genotype confirmation. Samples were sequenced using Big Dye Terminator v3.1 (AB, Foster City, CA, USA) according to the manufacturer’s guidelines and sequenced using an AB 3730 genetic analyzer.

**Table 1:** Polymorphisms within the *LRP1B* region in the CAA formation in Taiwanese Kawasaki disease.

SNP	SNP Chromosome	Cytoband	Physical Position	Nearest Genes		CAA-		CAA+	
						No. (%)	No. (%)	*p* value	Odds ratio (95% CI)
rs6742944	2	q22.1	139753322	*LRP1B*	AA+AG	47 (25.3)	12 (15.8)	0.099	0.55 (0.28-1.12)
					GG	139 (74.7)	64 (84.2)		1
rs6707826	2	q22.1	139793736	*LRP1B*	TT+TG	131 (70.4)	66 (86.8)	0.007*	2.77 (1.33-5.78)
					GG	55 (29.6)	10 (13.2)		1
rs17760152	2	q22.1	139795880	*LRP1B*	AA+AT	64 (35.8)	33 (45.2)	0.163	1.48 (0.85-2.58)
					TT	115 (64.2)	40 (54.8)		1
rs13400775	2	q22.1	139851962	*LRP1B*	GG+GC	66 (35.5)	33 (43.4)	0.230	1.4 (0.81-2.4)
					CC	120 (64.5)	43 (56.6)		1
rs7604667	2	q22.1	139899653	*LRP1B*	TT+TC	137 (73.7)	57 (75.0)	0.822	1.07 (0.58-1.98)
					CC	49 (26.3)	19 (25.0)		1

*LRP1B* , low density lipoprotein receptor-related protein 1B; SNP, single nucleotide polymorphism; CAA, Coronary artery aneurysm; CI, confidence interval. *p* -values were obtained by chi-square test

Bold, emphasizing statistical significance was considered as *p* value <0.010 (0.05/5).

### 2.4. Analysis of haplotype blocks

Based on HAPLOVIEW software, we used Lewontin D′ measure to estimate the intermarker coefficient of linkage disequilibrium (LD) of patients [23]. The confidence interval of LD was estimated using a resampling procedure and was used to construct haplotype blocks [25].

### 2.5. Statistical analysis

Statistical analyses were described previously[26]. Genotypes were obtained by direct counting followed by allele frequency calculations variables, and odds ratios (OR) and 95% confidence intervals (CI) were calculated for the factors under consideration. Forward stepwise multivariate regression analyses were also performed to identify factors contributing independently to CAA formation in KD (Table 2). All statistical analyses were performed using SPSS (v12.0) as described before [26].

## 3. Results

The genetic location of *LRP1B* was shown in Fig. 1; all SNPs were in Hardy-Weinberg equilibrium and had a successful genotyping frequency of >99%. The LD structure of this region was also established, with 1 haplotype block determined.

Genotype and genotype frequency data for all five SNPs were shown in Table 1. As shown, a statistically significant difference was observed for the *LRP1B* (rs6707826) genetic variant (*p* = 0.007). Frequencies of individuals carrying the TT+TG genotypes of *LRP1B* (rs6707826) were 86.8% for CAA-positive individuals in contrast to 70.4% for CAA-negative patients. The frequencies of TT and TG combined genotypes were significantly higher in KD with CAA formation (odds ratio = 2.77; 95% CI = 1.33-5.78) compared to those in KD without CAA formation.

To learn the genetic role of *LRP1B*, we used multivariate regression analyses to determine the associations of clinical characteristics and *LRP1B* genetic variations in KD. As shown in Table 2, after adjusting for those potential factors, significant associations between KD with CAA complication and the *LRP1B* (rs6707826) genetic variant was observed. Specifically, significant correlations were found between KD with CAA formation and the presence of the TT+TG genotypes of the *LRP1B* (rs6707826) SNP (Full model: odds ratio = 2.820; 95% CI = 1.33-5.78).

**Table 2:** Association of *LRP1* Bgenetic variants with CAA formation risk in Taiwanese Kawasaki disease by multivariate regression analysis.

*LRP1B*genetic variants	Odds ratio	95% CI	*P* value
**Adjusted by Fever duration (days)**			
rs6742944	0.71	0.28-1.12	0.353
rs6707826	2.77	1.35-5.78	0.010*
rs17760152	1.47	0.85-2.58	0.195
rs13400775	1.29	0.81-2.4	0.382
rs7604667	0.94	0.58-1.98	0.849
**Adjusted by 1st IVIG used time (days after the first date with fever)**			
rs6742944	0.55	0.28-1.12	0.099
rs6707826	2.720	1.33-5.78	0.008*
rs17760152	1.500	0.85-2.58	0.150
rs13400775	1.410	0.81-2.40	0.221
rs7604667	1.100	0.58-1.98	0.762
**Full Model**			
rs6742944	0.720	0.28-1.12	0.378
rs6707826	2.820	1.33-5.78	0.009*
rs17760152	1.280	0.81-2.4	0.405
rs13400775	0.910	0.58-1.98	0.788
rs7604667			

*LRP1B* , low density lipoprotein receptor-related protein 1B; IVIG, Intravenous immunoglobulin; CAA, Coronary artery aneurysm; CI, confidence interval. Full model shows results from a logistic regression model including the indicated predictors including Fever duration (days) and 1st IVIG used time (days after the first date with fever).

## 4. Discussion

In this study, we used a mapping strategy focusing on the *LRP1B* gene and identified a SNP that contributes to the development of CAA formation in Taiwanese KD children of Han Chinese ethnic background. We observed a significant association between the *LRP1B* gene polymorphism and the occurrence of CAA in KD patients by using multivariate regression analysis. The combined frequency of the TT+TG genotypes of *LRP1B* (rs6707826) was higher in KD with CAA group than those in KD without CAA group. Our results suggest that *LRP1B* polymorphism may play a role in the formation of CAA in KD patients.

The genetic association study showed that significant associations between KD with CAA formation and the *LRP1B* (rs6707826) were still observed by using multivariate regression analysis. The genotypic frequencies harboring one or two copies of T allele were higher in KD patients with CAA formation. These results suggest that the *LRP1B* gene polymorphism is involved in KD pathogenesis. Patients with one or more copies of T allele at the *LRP1B* gene tend to develop CAA. This genetic variant is located at the 3′UTR of the *LRP1B* gene and is putatively involved in posttranscriptional mRNA regulation. In addition, the SNP we identified (rs6707826) showed a linkage disequilibrium with the SNP (rs17760152) (Supplementary Table 1; D′ = 1). *LRP1B* expression has previously been shown to be significantly associated with the SNP (rs17760152) (*p* = 0.03996) (http://app3.titan.uio.no/biotools/tool.php?app=snpexp).

The low density lipoprotein receptor gene, *LRP1B* is located at chromosome 2q22.1 [27]. Its genomic DNA contains 91 exons and spans more than 500 kb. LRP1B protein has four putative ligand-binding domains which bind or endocytose different ligands including urokinase-type plasminogen activator (uPA), tissue-type plasminogen activator (tPA), plasminogen activator inhibitor-1 (PAI-1) and receptor-associated protein (RAP) [9]. In specimens of coronary arteries, LRP1B is clearly observed in intimal and medial smooth muscle cells [12]. The increased expression of LRP1B is shown to attenuate the migration of smooth muscle cells by reducing membrane localization of urokinase and platelet-derived growth factor (PDGF) receptors. LRP1B shares striking similarities to LRP1 protein. Knockout of *LRP1* have been also shown to increase the atherosclerotic lesion area and regulate inflammatory responses [28]. Furthermore, studies in the *LRP1* genetic variants are associated with an increased risk of coronary artery diseases [29-31]. KD is a multisystemic disorder with the possible underlying pathology of immune-mediated vasculitis and CAA complications [1, 32]. The roles of LRP1B receptor in regulating the vascular inflammation is yet to be investigated. Our data together suggest that *LRP1B* polymorphism may be involved in the formation of CAA in KD pathogenesis.

In conclusion, our results indicate that *LRP1B* is significantly associated with the CAA formation in KD progression in Taiwanese children with Han Chinese ethnic background. Genetic polymorphism of *LRP1B* gene may be used as a genetic marker for the diagnosis and prognosis of the CAA formation in KD.

## Acknowledgements

Support for this research was provided by CMU (CMU100-S-01), CMUH (DMR-103-100), and the Republic of China National Science Council (NSC 99-2628-B-039-001- MY3; NSC100-2320-B-039-012-MY3).
